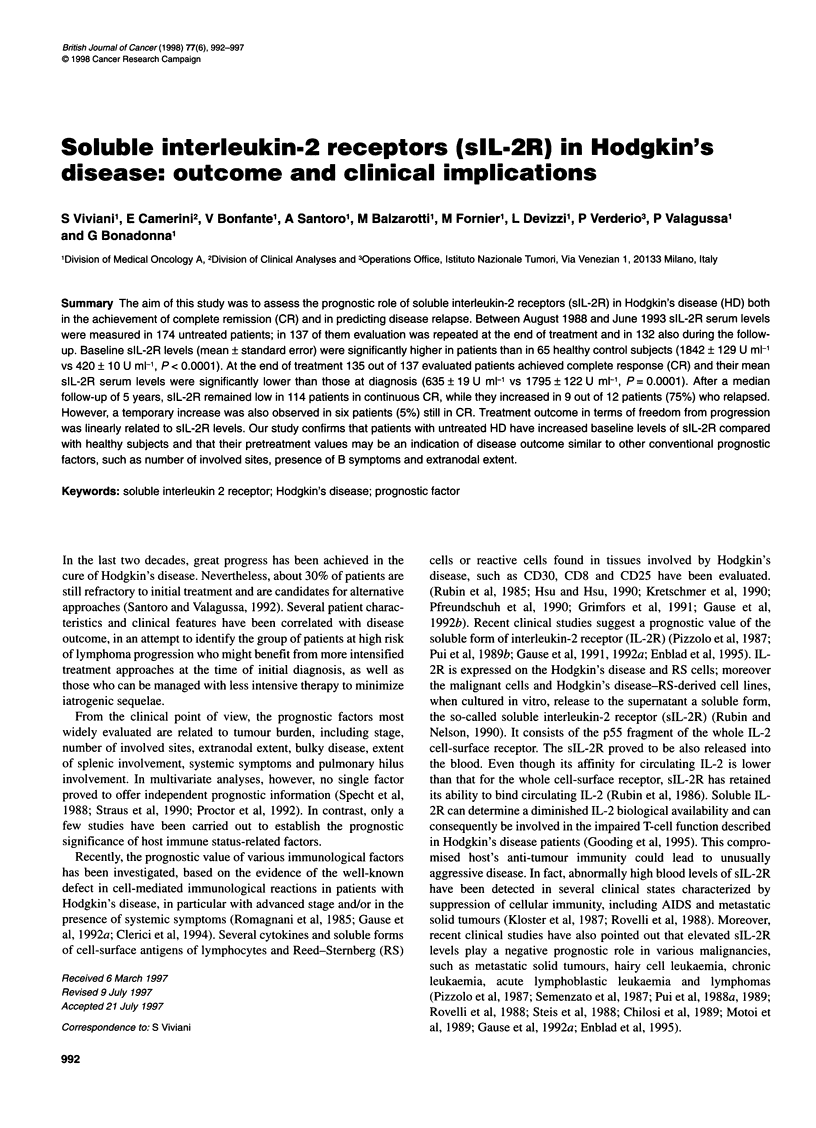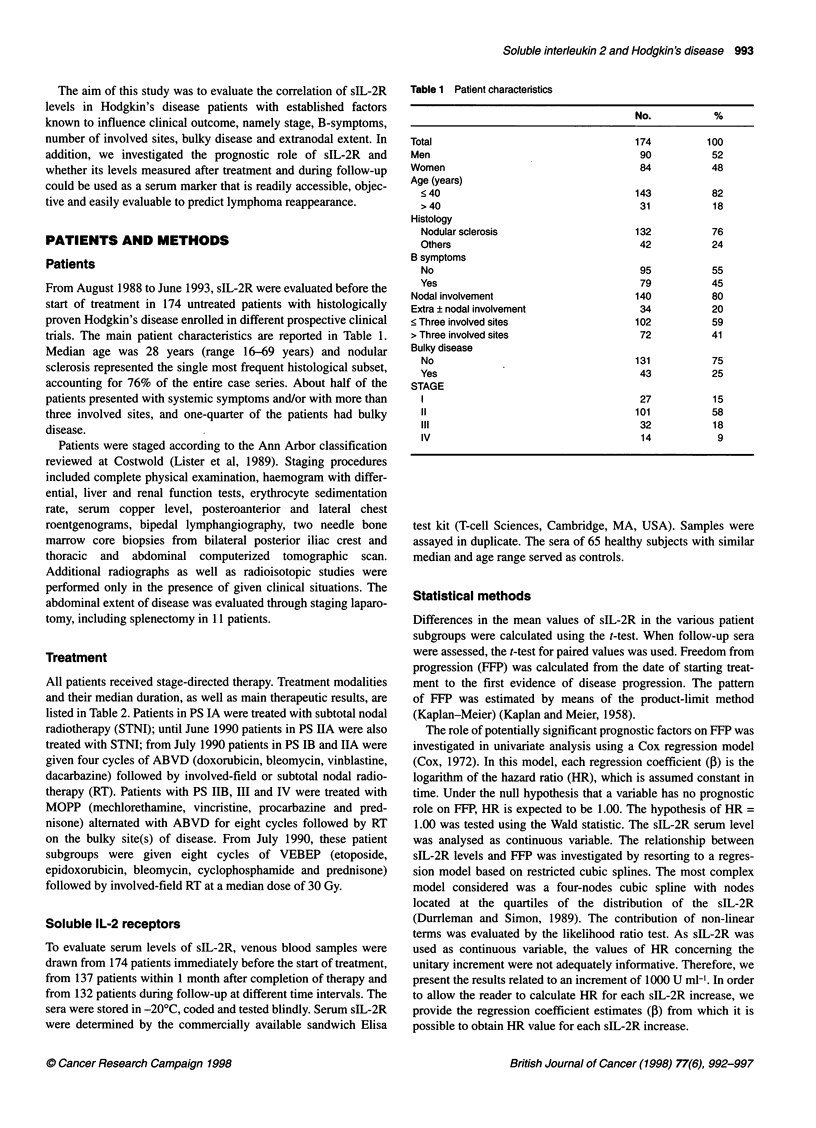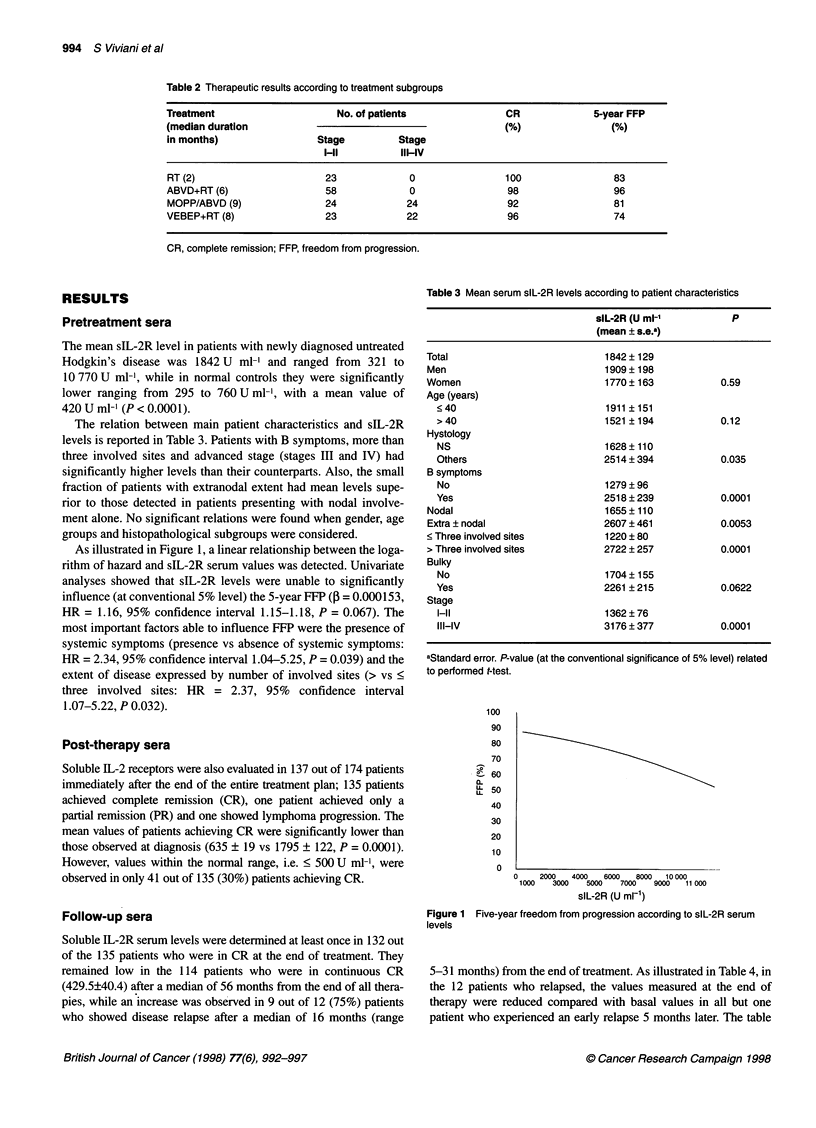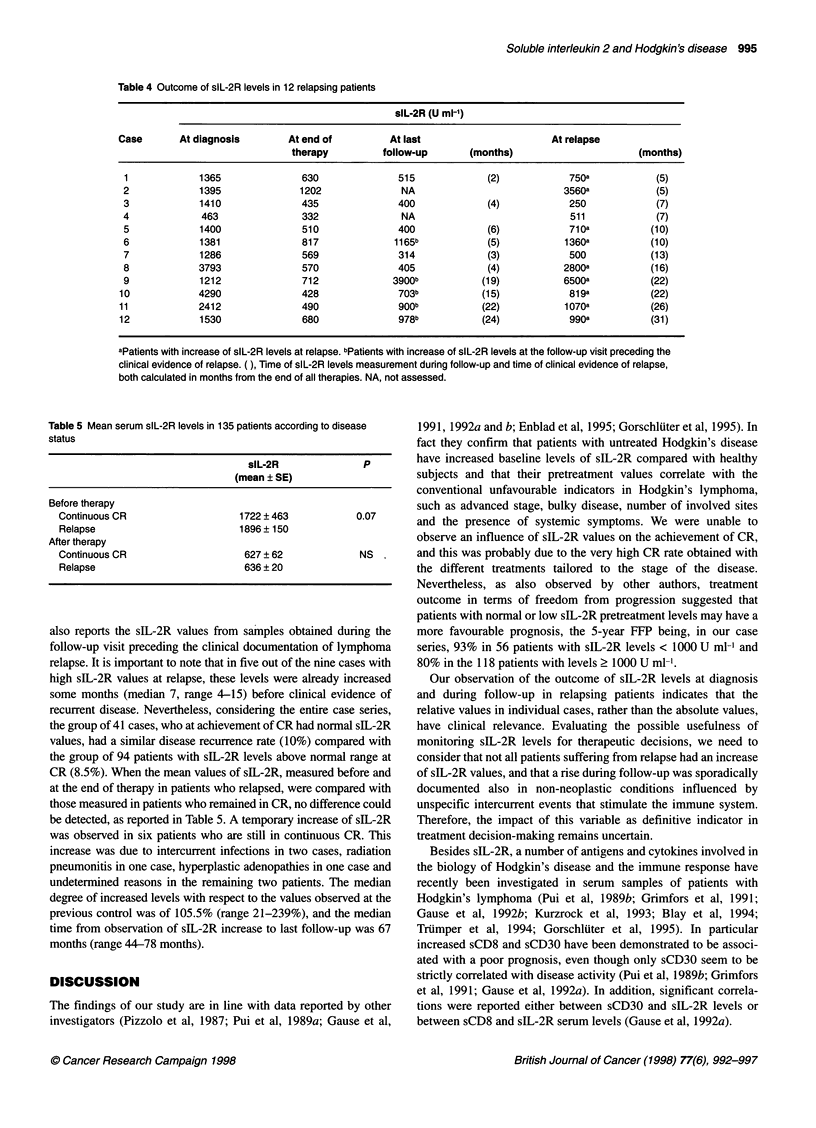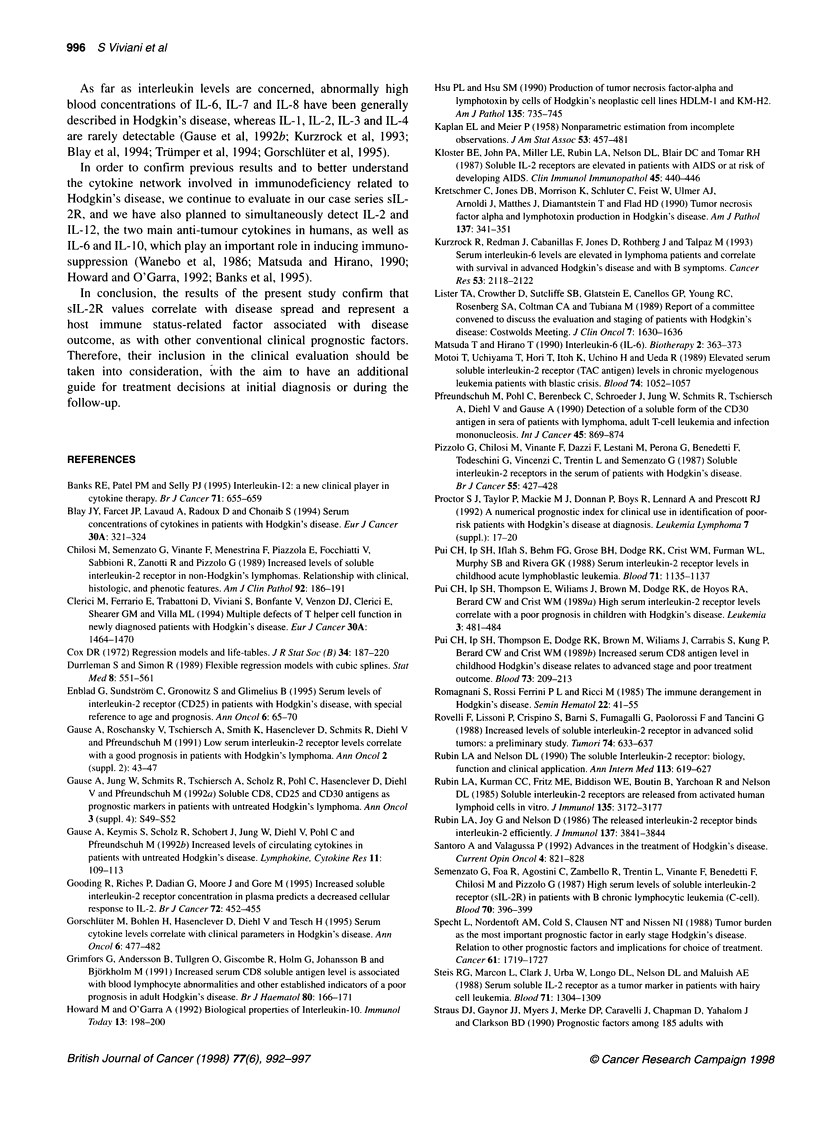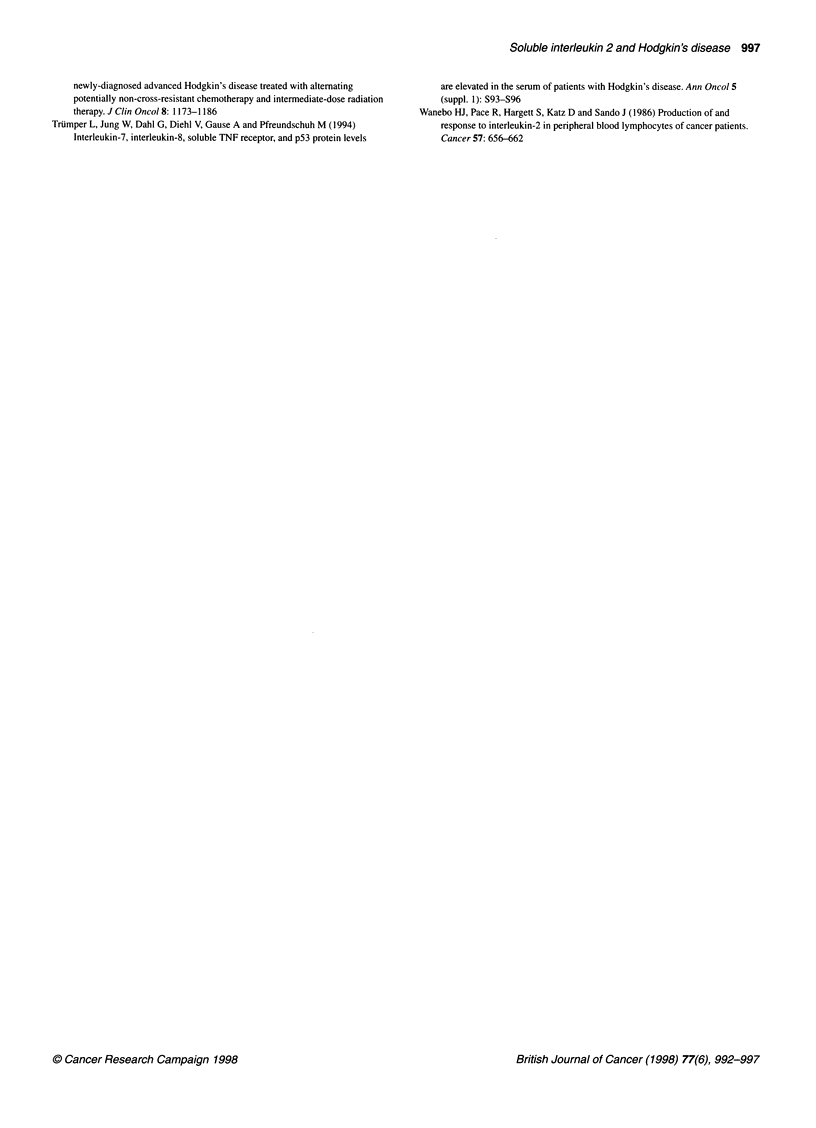# Soluble interleukin-2 receptors (sIL-2R) in Hodgkin's disease: outcome and clinical implications.

**DOI:** 10.1038/bjc.1998.163

**Published:** 1998-03

**Authors:** S. Viviani, E. Camerini, V. Bonfante, A. Santoro, M. Balzarotti, M. Fornier, L. Devizzi, P. Verderio, P. Valagussa, G. Bonadonna

**Affiliations:** Division of Medical Oncology A, Istituto Nazionale Tumori, Milano, Italy.

## Abstract

The aim of this study was to assess the prognostic role of soluble interleukin-2 receptors (sIL-2R) in Hodgkin's disease (HD) both in the achievement of complete remission (CR) and in predicting disease relapse. Between August 1988 and June 1993 sIL-2R serum levels were measured in 174 untreated patients; in 137 of them evaluation was repeated at the end of treatment and in 132 also during the follow-up. Baseline sIL-2R levels (mean+/-standard error) were significantly higher in patients than in 65 healthy control subjects (1842+/-129 U ml(-1) vs 420+/-10 U ml(-10, P< 0.0001). At the end of treatment 135 out of 137 evaluated patients achieved complete response (CR) and their mean sIL-2R serum levels were significantly lower than those at diagnosis (635+/-19 U ml(-1) vs 1795+/-122 U ml(-1), P=0.0001). After a median follow-up of 5 years, sIL-2R remained low in 114 patients in continuous CR, while they increased in 9 out of 12 patients (75%) who relapsed. However, a temporary increase was also observed in six patients (5%) still in CR. Treatment outcome in terms of freedom from progression was linearly related to sIL-2R levels. Our study confirms that patients with untreated HD have increased baseline levels of sIL-2R compared with healthy subjects and that their pretreatment values may be an indication of disease outcome similar to other conventional prognostic factors, such as number of involved sites, presence of B symptoms and extranodal extent.


British Journal of Cancer (1998) 77(6), 992–997
© 1998 Cancer Research Campaign

### Soluble interleukin-2 receptors (sIL-2R) in Hodgkin's disease: outcome and clinical implications

**S Viviani[1], E Camerini[2], V Bonfante[1], A Santoro[1], M Balzarotti[1], M Fornier[1], L Devizzi[1], P Verderio[3], P Valagussa[1] and G Bonadonna[1]**

[1]Division of Medical Oncology A, [2]Division of Clinical Analyses and [3]Operations Office, Istituto Nazionale Tumori, Via Venezian 1, 20133 Milano, Italy

**Summary** The aim of this study was to assess the prognostic role of soluble interleukin-2 receptors (sIL-2R) in Hodgkin's disease (HD) both in the achievement of complete remission (CR) and in predicting disease relapse. Between August 1988 and June 1993 sIL-2R serum levels were measured in 174 untreated patients; in 137 of them evaluation was repeated at the end of treatment and in 132 also during the follow-up. Baseline sIL-2R levels (mean ± standard error) were significantly higher in patients than in 65 healthy control subjects (1842 ± 129 U ml$^{-1}$ vs 420 ± 10 U ml$^{-1}$, $P < 0.0001$). At the end of treatment 135 out of 137 evaluated patients achieved complete response (CR) and their mean sIL-2R serum levels were significantly lower than those at diagnosis (635 ± 19 U ml$^{-1}$ vs 1795 ± 122 U ml$^{-1}$, $P = 0.0001$). After a median follow-up of 5 years, sIL-2R remained low in 114 patients in continuous CR, while they increased in 9 out of 12 patients (75%) who relapsed. However, a temporary increase was also observed in six patients (5%) still in CR. Treatment outcome in terms of freedom from progression was linearly related to sIL-2R levels. Our study confirms that patients with untreated HD have increased baseline levels of sIL-2R compared with healthy subjects and that their pretreatment values may be an indication of disease outcome similar to other conventional prognostic factors, such as number of involved sites, presence of B symptoms and extranodal extent.

**Keywords:** soluble interleukin 2 receptor; Hodgkin's disease; prognostic factor

In the last two decades, great progress has been achieved in the cure of Hodgkin's disease. Nevertheless, about 30% of patients are still refractory to initial treatment and are candidates for alternative approaches (Santoro and Valagussa, 1992). Several patient characteristics and clinical features have been correlated with disease outcome, in an attempt to identify the group of patients at high risk of lymphoma progression who might benefit from more intensified treatment approaches at the time of initial diagnosis, as well as those who can be managed with less intensive therapy to minimize iatrogenic sequelae.

From the clinical point of view, the prognostic factors most widely evaluated are related to tumour burden, including stage, number of involved sites, extranodal extent, bulky disease, extent of splenic involvement, systemic symptoms and pulmonary hilus involvement. In multivariate analyses, however, no single factor proved to offer independent prognostic information (Specht et al, 1988; Straus et al, 1990; Proctor et al, 1992). In contrast, only a few studies have been carried out to establish the prognostic significance of host immune status-related factors.

Recently, the prognostic value of various immunological factors has been investigated, based on the evidence of the well-known defect in cell-mediated immunological reactions in patients with Hodgkin's disease, in particular with advanced stage and/or in the presence of systemic symptoms (Romagnani et al, 1985; Gause et al, 1992*a*; Clerici et al, 1994). Several cytokines and soluble forms of cell-surface antigens of lymphocytes and Reed–Sternberg (RS) cells or reactive cells found in tissues involved by Hodgkin's disease, such as CD30, CD8 and CD25 have been evaluated. (Rubin et al, 1985; Hsu and Hsu, 1990; Kretschmer et al, 1990; Pfreundschuh et al, 1990; Grimfors et al, 1991; Gause et al, 1992*b*). Recent clinical studies suggest a prognostic value of the soluble form of interleukin-2 receptor (IL-2R) (Pizzolo et al, 1987; Pui et al, 1989*b*; Gause et al, 1991, 1992*a*; Enblad et al, 1995). IL-2R is expressed on the Hodgkin's disease and RS cells; moreover the malignant cells and Hodgkin's disease–RS-derived cell lines, when cultured in vitro, release to the supernatant a soluble form, the so-called soluble interleukin-2 receptor (sIL-2R) (Rubin and Nelson, 1990). It consists of the p55 fragment of the whole IL-2 cell-surface receptor. The sIL-2R proved to be also released into the blood. Even though its affinity for circulating IL-2 is lower than that for the whole cell-surface receptor, sIL-2R has retained its ability to bind circulating IL-2 (Rubin et al, 1986). Soluble IL-2R can determine a diminished IL-2 biological availability and can consequently be involved in the impaired T-cell function described in Hodgkin's disease patients (Gooding et al, 1995). This compromised host's anti-tumour immunity could lead to unusually aggressive disease. In fact, abnormally high blood levels of sIL-2R have been detected in several clinical states characterized by suppression of cellular immunity, including AIDS and metastatic solid tumours (Kloster et al, 1987; Rovelli et al, 1988). Moreover, recent clinical studies have also pointed out that elevated sIL-2R levels play a negative prognostic role in various malignancies, such as metastatic solid tumours, hairy cell leukaemia, chronic leukaemia, acute lymphoblastic leukaemia and lymphomas (Pizzolo et al, 1987; Semenzato et al, 1987; Pui et al, 1988*a*, 1989; Rovelli et al, 1988; Steis et al, 1988; Chilosi et al, 1989; Motoi et al, 1989; Gause et al, 1992*a*; Enblad et al, 1995).

*Received 6 March 1997*
*Revised 9 July 1997*
*Accepted 21 July 1997*

*Correspondence to:* S Viviani

The aim of this study was to evaluate the correlation of sIL-2R levels in Hodgkin's disease patients with established factors known to influence clinical outcome, namely stage, B-symptoms, number of involved sites, bulky disease and extranodal extent. In addition, we investigated the prognostic role of sIL-2R and whether its levels measured after treatment and during follow-up could be used as a serum marker that is readily accessible, objective and easily evaluable to predict lymphoma reappearance.

### PATIENTS AND METHODS

### Patients

From August 1988 to June 1993, sIL-2R were evaluated before the start of treatment in 174 untreated patients with histologically proven Hodgkin's disease enrolled in different prospective clinical trials. The main patient characteristics are reported in Table 1. Median age was 28 years (range 16–69 years) and nodular sclerosis represented the single most frequent histological subset, accounting for 76% of the entire case series. About half of the patients presented with systemic symptoms and/or with more than three involved sites, and one-quarter of the patients had bulky disease.

Patients were staged according to the Ann Arbor classification reviewed at Costwold (Lister et al, 1989). Staging procedures included complete physical examination, haemogram with differential, liver and renal function tests, erythrocyte sedimentation rate, serum copper level, posteroanterior and lateral chest roentgenograms, bipedal lymphangiography, two needle bone marrow core biopsies from bilateral posterior iliac crest and thoracic and abdominal computerized tomographic scan. Additional radiographs as well as radioisotopic studies were performed only in the presence of given clinical situations. The abdominal extent of disease was evaluated through staging laparotomy, including splenectomy in 11 patients.

### Treatment

All patients received stage-directed therapy. Treatment modalities and their median duration, as well as main therapeutic results, are listed in Table 2. Patients in PS IA were treated with subtotal nodal radiotherapy (STNI); until June 1990 patients in PS IIA were also treated with STNI; from July 1990 patients in PS IB and IIA were given four cycles of ABVD (doxorubicin, bleomycin, vinblastine, dacarbazine) followed by involved-field or subtotal nodal radiotherapy (RT). Patients with PS IIB, III and IV were treated with MOPP (mechlorethamine, vincristine, procarbazine and prednisone) alternated with ABVD for eight cycles followed by RT on the bulky site(s) of disease. From July 1990, these patient subgroups were given eight cycles of VEBEP (etoposide, epidoxorubicin, bleomycin, cyclophosphamide and prednisone) followed by involved-field RT at a median dose of 30 Gy.

### Soluble IL-2 receptors

To evaluate serum levels of sIL-2R, venous blood samples were drawn from 174 patients immediately before the start of treatment, from 137 patients within 1 month after completion of therapy and from 132 patients during follow-up at different time intervals. The sera were stored in –20°C, coded and tested blindly. Serum sIL-2R were determined by the commercially available sandwich Elisa test kit (T-cell Sciences, Cambridge, MA, USA). Samples were assayed in duplicate. The sera of 65 healthy subjects with similar median and age range served as controls.

**Table 1** Patient characteristics

| | No. | % |
|---|---|---|
| Total | 174 | 100 |
| Men | 90 | 52 |
| Women | 84 | 48 |
| Age (years) | | |
| ≤ 40 | 143 | 82 |
| > 40 | 31 | 18 |
| Histology | | |
| Nodular sclerosis | 132 | 76 |
| Others | 42 | 24 |
| B symptoms | | |
| No | 95 | 55 |
| Yes | 79 | 45 |
| Nodal involvement | 140 | 80 |
| Extra ± nodal involvement | 34 | 20 |
| ≤ Three involved sites | 102 | 59 |
| > Three involved sites | 72 | 41 |
| Bulky disease | | |
| No | 131 | 75 |
| Yes | 43 | 25 |
| STAGE | | |
| I | 27 | 15 |
| II | 101 | 58 |
| III | 32 | 18 |
| IV | 14 | 9 |

### Statistical methods

Differences in the mean values of sIL-2R in the various patient subgroups were calculated using the *t*-test. When follow-up sera were assessed, the *t*-test for paired values was used. Freedom from progression (FFP) was calculated from the date of starting treatment to the first evidence of disease progression. The pattern of FFP was estimated by means of the product-limit method (Kaplan–Meier) (Kaplan and Meier, 1958).

The role of potentially significant prognostic factors on FFP was investigated in univariate analysis using a Cox regression model (Cox, 1972). In this model, each regression coefficient (β) is the logarithm of the hazard ratio (HR), which is assumed constant in time. Under the null hypothesis that a variable has no prognostic role on FFP, HR is expected to be 1.00. The hypothesis of HR = 1.00 was tested using the Wald statistic. The sIL-2R serum level was analysed as continuous variable. The relationship between sIL-2R levels and FFP was investigated by resorting to a regression model based on restricted cubic splines. The most complex model considered was a four-nodes cubic spline with nodes located at the quartiles of the distribution of the sIL-2R (Durrleman and Simon, 1989). The contribution of non-linear terms was evaluated by the likelihood ratio test. As sIL-2R was used as continuous variable, the values of HR concerning the unitary increment were not adequately informative. Therefore, we present the results related to an increment of 1000 U $ml^{-1}$. In order to allow the reader to calculate HR for each sIL-2R increase, we provide the regression coefficient estimates (β) from which it is possible to obtain HR value for each sIL-2R increase.

© Cancer Research Campaign 1998

Table 2 Therapeutic results according to treatment subgroups

| Treatment (median duration in months) | No. of patients | | CR (%) | 5-year FFP (%) |
|---|---|---|---|---|
| | Stage I–II | Stage III–IV | | |
| RT (2) | 23 | 0 | 100 | 83 |
| ABVD+RT (6) | 58 | 0 | 98 | 96 |
| MOPP/ABVD (9) | 24 | 24 | 92 | 81 |
| VEBEP+RT (8) | 23 | 22 | 96 | 74 |

CR, complete remission; FFP, freedom from progression.

### RESULTS

### Pretreatment sera

The mean sIL-2R level in patients with newly diagnosed untreated Hodgkin's disease was 1842 U ml$^{-1}$ and ranged from 321 to 10 770 U ml$^{-1}$, while in normal controls they were significantly lower ranging from 295 to 760 U ml$^{-1}$, with a mean value of 420 U ml$^{-1}$ ($P < 0.0001$).

The relation between main patient characteristics and sIL-2R levels is reported in Table 3. Patients with B symptoms, more than three involved sites and advanced stage (stages III and IV) had significantly higher levels than their counterparts. Also, the small fraction of patients with extranodal extent had mean levels superior to those detected in patients presenting with nodal involvement alone. No significant relations were found when gender, age groups and histopathological subgroups were considered.

As illustrated in Figure 1, a linear relationship between the logarithm of hazard and sIL-2R serum values was detected. Univariate analyses showed that sIL-2R levels were unable to significantly influence (at conventional 5% level) the 5-year FFP ($\beta = 0.000153$, HR = 1.16, 95% confidence interval 1.15–1.18, $P = 0.067$). The most important factors able to influence FFP were the presence of systemic symptoms (presence vs absence of systemic symptoms: HR = 2.34, 95% confidence interval 1.04–5.25, $P = 0.039$) and the extent of disease expressed by number of involved sites (> vs ≤ three involved sites: HR = 2.37, 95% confidence interval 1.07–5.22, $P$ 0.032).

### Post-therapy sera

Soluble IL-2 receptors were also evaluated in 137 out of 174 patients immediately after the end of the entire treatment plan; 135 patients achieved complete remission (CR), one patient achieved only a partial remission (PR) and one showed lymphoma progression. The mean values of patients achieving CR were significantly lower than those observed at diagnosis (635 ± 19 vs 1795 ± 122, $P = 0.0001$). However, values within the normal range, i.e. ≤ 500 U ml$^{-1}$, were observed in only 41 out of 135 (30%) patients achieving CR.

### Follow-up sera

Soluble IL-2R serum levels were determined at least once in 132 out of the 135 patients who were in CR at the end of treatment. They remained low in the 114 patients who were in continuous CR (429.5±40.4) after a median of 56 months from the end of all therapies, while an increase was observed in 9 out of 12 (75%) patients who showed disease relapse after a median of 16 months (range 5–31 months) from the end of treatment. As illustrated in Table 4, in the 12 patients who relapsed, the values measured at the end of therapy were reduced compared with basal values in all but one patient who experienced an early relapse 5 months later. The table

Table 3 Mean serum sIL-2R levels according to patient characteristics

| | sIL-2R (U ml$^{-1}$ (mean ± s.e.[a]) | P |
|---|---|---|
| Total | 1842 ± 129 | |
| Men | 1909 ± 198 | |
| Women | 1770 ± 163 | 0.59 |
| Age (years) | | |
| ≤ 40 | 1911 ± 151 | |
| > 40 | 1521 ± 194 | 0.12 |
| Hystology | | |
| NS | 1628 ± 110 | |
| Others | 2514 ± 394 | 0.035 |
| B symptoms | | |
| No | 1279 ± 96 | |
| Yes | 2518 ± 239 | 0.0001 |
| Nodal | 1655 ± 110 | |
| Extra ± nodal | 2607 ± 461 | 0.0053 |
| ≤ Three involved sites | 1220 ± 80 | |
| > Three involved sites | 2722 ± 257 | 0.0001 |
| Bulky | | |
| No | 1704 ± 155 | |
| Yes | 2261 ± 215 | 0.0622 |
| Stage | | |
| I–II | 1362 ± 76 | |
| III–IV | 3176 ± 377 | 0.0001 |

[a]Standard error. P-value (at the conventional significance of 5% level) related to performed t-test.

Figure 1 Five-year freedom from progression according to sIL-2R serum levels

© Cancer Research Campaign 1998

**Table 4** Outcome of sIL-2R levels in 12 relapsing patients

| Case | sIL-2R (U ml$^{-1}$) | | | | | |
|---|---|---|---|---|---|---|
| | At diagnosis | At end of therapy | At last follow-up | (months) | At relapse | (months) |
| 1 | 1365 | 630 | 515 | (2) | 750[a] | (5) |
| 2 | 1395 | 1202 | NA | | 3560[a] | (5) |
| 3 | 1410 | 435 | 400 | (4) | 250 | (7) |
| 4 | 463 | 332 | NA | | 511 | (7) |
| 5 | 1400 | 510 | 400 | (6) | 710[a] | (10) |
| 6 | 1381 | 817 | 1165[b] | (5) | 1360[a] | (10) |
| 7 | 1286 | 569 | 314 | (3) | 500 | (13) |
| 8 | 3793 | 570 | 405 | (4) | 2800[a] | (16) |
| 9 | 1212 | 712 | 3900[b] | (19) | 6500[a] | (22) |
| 10 | 4290 | 428 | 703[b] | (15) | 819[a] | (22) |
| 11 | 2412 | 490 | 900[b] | (22) | 1070[a] | (26) |
| 12 | 1530 | 680 | 978[b] | (24) | 990[a] | (31) |

[a]Patients with increase of sIL-2R levels at relapse. [b]Patients with increase of sIL-2R levels at the follow-up visit preceding the clinical evidence of relapse. ( ), Time of sIL-2R levels measurement during follow-up and time of clinical evidence of relapse, both calculated in months from the end of all therapies. NA, not assessed.

**Table 5** Mean serum sIL-2R levels in 135 patients according to disease status

| | sIL-2R (mean ± SE) | *P* |
|---|---|---|
| Before therapy | | |
| Continuous CR | 1722 ± 463 | 0.07 |
| Relapse | 1896 ± 150 | |
| After therapy | | |
| Continuous CR | 627 ± 62 | NS |
| Relapse | 636 ± 20 | |

also reports the sIL-2R values from samples obtained during the follow-up visit preceding the clinical documentation of lymphoma relapse. It is important to note that in five out of the nine cases with high sIL-2R values at relapse, these levels were already increased some months (median 7, range 4–15) before clinical evidence of recurrent disease. Nevertheless, considering the entire case series, the group of 41 cases, who at achievement of CR had normal sIL-2R values, had a similar disease recurrence rate (10%) compared with the group of 94 patients with sIL-2R levels above normal range at CR (8.5%). When the mean values of sIL-2R, measured before and at the end of therapy in patients who relapsed, were compared with those measured in patients who remained in CR, no difference could be detected, as reported in Table 5. A temporary increase of sIL-2R was observed in six patients who are still in continuous CR. This increase was due to intercurrent infections in two cases, radiation pneumonitis in one case, hyperplastic adenopathies in one case and undetermined reasons in the remaining two patients. The median degree of increased levels with respect to the values observed at the previous control was of 105.5% (range 21–239%), and the median time from observation of sIL-2R increase to last follow-up was 67 months (range 44–78 months).

### DISCUSSION

The findings of our study are in line with data reported by other investigators (Pizzolo et al, 1987; Pui et al, 1989*a*; Gause et al, 1991, 1992*a* and *b*; Enblad et al, 1995; Gorschlüter et al, 1995). In fact they confirm that patients with untreated Hodgkin's disease have increased baseline levels of sIL-2R compared with healthy subjects and that their pretreatment values correlate with the conventional unfavourable indicators in Hodgkin's lymphoma, such as advanced stage, bulky disease, number of involved sites and the presence of systemic symptoms. We were unable to observe an influence of sIL-2R values on the achievement of CR, and this was probably due to the very high CR rate obtained with the different treatments tailored to the stage of the disease. Nevertheless, as also observed by other authors, treatment outcome in terms of freedom from progression suggested that patients with normal or low sIL-2R pretreatment levels may have a more favourable prognosis, the 5-year FFP being, in our case series, 93% in 56 patients with sIL-2R levels < 1000 U ml$^{-1}$ and 80% in the 118 patients with levels ≥ 1000 U ml$^{-1}$.

Our observation of the outcome of sIL-2R levels at diagnosis and during follow-up in relapsing patients indicates that the relative values in individual cases, rather than the absolute values, have clinical relevance. Evaluating the possible usefulness of monitoring sIL-2R levels for therapeutic decisions, we need to consider that not all patients suffering from relapse had an increase of sIL-2R values, and that a rise during follow-up was sporadically documented also in non-neoplastic conditions influenced by unspecific intercurrent events that stimulate the immune system. Therefore, the impact of this variable as definitive indicator in treatment decision-making remains uncertain.

Besides sIL-2R, a number of antigens and cytokines involved in the biology of Hodgkin's disease and the immune response have recently been investigated in serum samples of patients with Hodgkin's lymphoma (Pui et al, 1989*b*; Grimfors et al, 1991; Gause et al, 1992*b*; Kurzrock et al, 1993; Blay et al, 1994; Trümper et al, 1994; Gorschlüter et al, 1995). In particular increased sCD8 and sCD30 have been demonstrated to be associated with a poor prognosis, even though only sCD30 seem to be strictly correlated with disease activity (Pui et al, 1989*b*; Grimfors et al, 1991; Gause et al, 1992*a*). In addition, significant correlations were reported either between sCD30 and sIL-2R levels or between sCD8 and sIL-2R serum levels (Gause et al, 1992*a*).

© Cancer Research Campaign 1998

As far as interleukin levels are concerned, abnormally high blood concentrations of IL-6, IL-7 and IL-8 have been generally described in Hodgkin's disease, whereas IL-1, IL-2, IL-3 and IL-4 are rarely detectable (Gause et al, 1992*b*; Kurzrock et al, 1993; Blay et al, 1994; Trümper et al, 1994; Gorschlüter et al, 1995).

In order to confirm previous results and to better understand the cytokine network involved in immunodeficiency related to Hodgkin's disease, we continue to evaluate in our case series sIL-2R, and we have also planned to simultaneously detect IL-2 and IL-12, the two main anti-tumour cytokines in humans, as well as IL-6 and IL-10, which play an important role in inducing immunosuppression (Wanebo et al, 1986; Matsuda and Hirano, 1990; Howard and O'Garra, 1992; Banks et al, 1995).

In conclusion, the results of the present study confirm that sIL-2R values correlate with disease spread and represent a host immune status-related factor associated with disease outcome, as with other conventional clinical prognostic factors. Therefore, their inclusion in the clinical evaluation should be taken into consideration, with the aim to have an additional guide for treatment decisions at initial diagnosis or during the follow-up.

### REFERENCES

Banks RE, Patel PM and Selly PJ (1995) Interleukin-12: a new clinical player in cytokine therapy. *Br J Cancer* **71**: 655–659

Blay JY, Farcet JP, Lavaud A, Radoux D and Chonaib S (1994) Serum concentrations of cytokines in patients with Hodgkin's disease. *Eur J Cancer* **30A**: 321–324

Chilosi M, Semenzato G, Vinante F, Menestrina F, Piazzola E, Focchiatti V, Sabbioni R, Zanotti R and Pizzolo G (1989) Increased levels of soluble interleukin-2 receptor in non-Hodgkin's lymphomas. Relationship with clinical, histologic, and phenotic features. *Am J Clin Pathol* **92**: 186–191

Clerici M, Ferrario E, Trabattoni D, Viviani S, Bonfante V, Venzon DJ, Clerici E, Shearer GM and Villa ML (1994) Multiple defects of T helper cell function in newly diagnosed patients with Hodgkin's disease. *Eur J Cancer* **30A**: 1464–1470

Cox DR (1972) Regression models and life-tables. *J R Stat Soc (B)* **34**: 187–220

Durrleman S and Simon R (1989) Flexible regression models with cubic splines. *Stat Med* **8**: 551–561

Enblad G, Sundström C, Gronowitz S and Glimelius B (1995) Serum levels of interleukin-2 receptor (CD25) in patients with Hodgkin's disease, with special reference to age and prognosis. *Ann Oncol* **6**: 65–70

Gause A, Roschansky V, Tschiersch A, Smith K, Hasenclever D, Schmits R, Diehl V and Pfreundschuh M (1991) Low serum interleukin-2 receptor levels correlate with a good prognosis in patients with Hodgkin's lymphoma. *Ann Oncol* **2** (suppl. 2): 43–47

Gause A, Jung W, Schmits R, Tschiersch A, Scholz R, Pohl C, Hasenclever D, Diehl V and Pfreundschuh M (1992*a*) Soluble CD8, CD25 and CD30 antigens as prognostic markers in patients with untreated Hodgkin's lymphoma. *Ann Oncol* **3** (suppl. 4): S49–S52

Gause A, Keymis S, Scholz R, Schobert J, Jung W, Diehl V, Pohl C and Pfreundschuh M (1992*b*) Increased levels of circulating cytokines in patients with untreated Hodgkin's disease. *Lymphokine, Cytokine Res* **11**: 109–113

Gooding R, Riches P, Dadian G, Moore J and Gore M (1995) Increased soluble interleukin-2 receptor concentration in plasma predicts a decreased cellular response to IL-2. *Br J Cancer* **72**: 452–455

Gorschlüter M, Bohlen H, Hasenclever D, Diehl V and Tesch H (1995) Serum cytokine levels correlate with clinical parameters in Hodgkin's disease. *Ann Oncol* **6**: 477–482

Grimfors G, Andersson B, Tullgren O, Giscombe R, Holm G, Johansson B and Björkholm M (1991) Increased serum CD8 soluble antigen level is associated with blood lymphocyte abnormalities and other established indicators of a poor prognosis in adult Hodgkin's disease. *Br J Haematol* **80**: 166–171

Howard M and O'Garra A (1992) Biological properties of Interleukin-10. *Immunol Today* **13**: 198–200

Hsu PL and Hsu SM (1990) Production of tumor necrosis factor-alpha and lymphotoxin by cells of Hodgkin's neoplastic cell lines HDLM-1 and KM-H2. *Am J Pathol* **135**: 735–745

Kaplan EL and Meier P (1958) Nonparametric estimation from incomplete observations. *J Am Stat Assoc* **53**: 457–481

Kloster BE, John PA, Miller LE, Rubin LA, Nelson DL, Blair DC and Tomar RH (1987) Soluble IL-2 receptors are elevated in patients with AIDS or at risk of developing AIDS. *Clin Immunol Immunopathol* **45**: 440–446

Kretschmer C, Jones DB, Morrison K, Schluter C, Feist W, Ulmer AJ, Arnoldi J, Matthes J, Diamantstein T and Flad HD (1990) Tumor necrosis factor alpha and lymphotoxin production in Hodgkin's disease. *Am J Pathol* **137**: 341–351

Kurzrock R, Redman J, Cabanillas F, Jones D, Rothberg J and Talpaz M (1993) Serum interleukin-6 levels are elevated in lymphoma patients and correlate with survival in advanced Hodgkin's disease and with B symptoms. *Cancer Res* **53**: 2118–2122

Lister TA, Crowther D, Sutcliffe SB, Glatstein E, Canellos GP, Young RC, Rosenberg SA, Coltman CA and Tubiana M (1989) Report of a committee convened to discuss the evaluation and staging of patients with Hodgkin's disease: Costwolds Meeting. *J Clin Oncol* **7**: 1630–1636

Matsuda T and Hirano T (1990) Interleukin-6 (IL-6). *Biotherapy* **2**: 363–373

Motoi T, Uchiyama T, Hori T, Itoh K, Uchino H and Ueda R (1989) Elevated serum soluble interleukin-2 receptor (TAC antigen) levels in chronic myelogenous leukemia patients with blastic crisis. *Blood* **74**: 1052–1057

Pfreundschuh M, Pohl C, Berenbeck C, Schroeder J, Jung W, Schmits R, Tschiersch A, Diehl V and Gause A (1990) Detection of a soluble form of the CD30 antigen in sera of patients with lymphoma, adult T-cell leukemia and infection mononucleosis. *Int J Cancer* **45**: 869–874

Pizzolo G, Chilosi M, Vinante F, Dazzi F, Lestani M, Perona G, Benedetti F, Todeschini G, Vincenzi C, Trentin L and Semenzato G (1987) Soluble interleukin-2 receptors in the serum of patients with Hodgkin's disease. *Br J Cancer* **55**: 427–428

Proctor S J, Taylor P, Mackie M J, Donnan P, Boys R, Lennard A and Prescott RJ (1992) A numerical prognostic index for clinical use in identification of poor-risk patients with Hodgkin's disease at diagnosis. *Leukemia Lymphoma* **7** (suppl.): 17–20

Pui CH, Ip SH, Iflah S, Behm FG, Grose BH, Dodge RK, Crist WM, Furman WL, Murphy SB and Rivera GK (1988) Serum interleukin-2 receptor levels in childhood acute lymphoblastic leukemia. *Blood* **71**: 1135–1137

Pui CH, Ip SH, Thompson E, Wiliams J, Brown M, Dodge RK, de Hoyos RA, Berard CW and Crist WM (1989*a*) High serum interleukin-2 receptor levels correlate with a poor prognosis in children with Hodgkin's disease. *Leukemia* **3**: 481–484

Pui CH, Ip SH, Thompson E, Dodge RK, Brown M, Wiliams J, Carrabis S, Kung P, Berard CW and Crist WM (1989*b*) Increased serum CD8 antigen level in childhood Hodgkin's disease relates to advanced stage and poor treatment outcome. *Blood* **73**: 209–213

Romagnani S, Rossi Ferrini P L and Ricci M (1985) The immune derangement in Hodgkin's disease. *Semin Hematol* **22**: 41–55

Rovelli F, Lissoni P, Crispino S, Barni S, Fumagalli G, Paolorossi F and Tancini G (1988) Increased levels of soluble interleukin-2 receptor in advanced solid tumors: a preliminary study. *Tumori* **74**: 633–637

Rubin LA and Nelson DL (1990) The soluble Interleukin-2 receptor: biology, function and clinical application. *Ann Intern Med* **113**: 619–627

Rubin LA, Kurman CC, Fritz ME, Biddison WE, Boutin B, Yarchoan R and Nelson DL (1985) Soluble interleukin-2 receptors are released from activated human lymphoid cells in vitro. *J Immunol* **135**: 3172–3177

Rubin LA, Joy G and Nelson D (1986) The released interleukin-2 receptor binds interleukin-2 efficiently. *J Immunol* **137**: 3841–3844

Santoro A and Valagussa P (1992) Advances in the treatment of Hodgkin's disease. *Current Opin Oncol* **4**: 821–828

Semenzato G, Foa R, Agostini C, Zambello R, Trentin L, Vinante F, Benedetti F, Chilosi M and Pizzolo G (1987) High serum levels of soluble interleukin-2 receptor (sIL-2R) in patients with B chronic lymphocytic leukemia (C-cell). *Blood* **70**: 396–399

Specht L, Nordentoft AM, Cold S, Clausen NT and Nissen NI (1988) Tumor burden as the most important prognostic factor in early stage Hodgkin's disease. Relation to other prognostic factors and implications for choice of treatment. *Cancer* **61**: 1719–1727

Steis RG, Marcon L, Clark J, Urba W, Longo DL, Nelson DL and Maluish AE (1988) Serum soluble IL-2 receptor as a tumor marker in patients with hairy cell leukemia. *Blood* **71**: 1304–1309

Straus DJ, Gaynor JJ, Myers J, Merke DP, Caravelli J, Chapman D, Yahalom J and Clarkson BD (1990) Prognostic factors among 185 adults with

© Cancer Research Campaign 1998

newly-diagnosed advanced Hodgkin's disease treated with alternating potentially non-cross-resistant chemotherapy and intermediate-dose radiation therapy. *J Clin Oncol* **8**: 1173–1186

Trümper L, Jung W, Dahl G, Diehl V, Gause A and Pfreundschuh M (1994) Interleukin-7, interleukin-8, soluble TNF receptor, and p53 protein levels are elevated in the serum of patients with Hodgkin's disease. *Ann Oncol* **5** (suppl. 1): S93–S96

Wanebo HJ, Pace R, Hargett S, Katz D and Sando J (1986) Production of and response to interleukin-2 in peripheral blood lymphocytes of cancer patients. *Cancer* **57**: 656–662

© Cancer Research Campaign 1998